# Criticism of Dr. Wilson’s Rejoinder to Dr. Wells on Vital Force

**Published:** 1884-04

**Authors:** B. Fincke

**Affiliations:** Brooklyn, N. Y.


### HOMŒOPATHIC PHYSICIAN,

A MONTHLY JOURNAL OF MEDICAL SCIENCE.

"If our school ever gives up the strict inductive method of Hahnemann, we are lost, and deserve only to be mentioned as a caricature in the history of medicine."—CONSTANTINE HERING.

Vol. IV. APRIL, 1884. No. 4.

### CRITICISM OF DR. WILSON'S REJOINDER TO DR. WELLS ON THE VITAL FORCE.

B. FINCKE, M.D., BROOKLYN, N. Y.

"*Allowing all he says to be true, it reduces him to the absurdity of attempting to prove a self-evident proposition.*"

But Dr. Wilson does not allow "all he says to be true;" on the contrary, he denies the vital force to be a self-evident proposition, and this makes the difference. He says, with the scientists, there is no such thing as a vital force. Hence it is perfectly right in Dr. Wells to demonstrate the self-evident truth that there is a vital force. Suppose Dr. Wilson were born blind, and Dr. Wells would tell him the self-evident truth that the sun shines, would he be justified to deny it on the ground that he is blind? No more is he entitled to deny, being intelligently blinded by the scientists of to-day, the vital force.

*When the point at issue is assumed by either party, to say the least, it cuts out the party so assuming, and the argument is ended.*

But the point at issue is not assumed by either party because Dr. Wilson denies the vital force to be self-evident. It, therefore, makes him liable to the argument applied to him.

*Since to our mind the question is debatable and worthy of study, we cannot follow Dr. Wells, etc.*

This is the loop-hole which proves the trap for Dr. Wilson. For it was Dr. Wilson who denied the existence of the vital force, being out of date to-day, and Dr. Wells maintained it and proposed to discuss it. If, then, Dr. Wilson finds the question worthy of debate, he is in accordance with Dr. Wells. Why, then, blame him with an absurdity which does not exist? Dr. Wilson therefore admits the vital force to be a proper subject for controversy, and contradicts himself.

*The facts of Homœopathy are one thing, and the theories which help to explain them are another thing.*

Just so. The standpoint of Dr. Wilson, which he stated admirably at the close of his editorial in the *Medical Advance*, November, 1883, does not leave any doubt of pure empiricism when he says: "Homœopathy is a simple, straightforward system of therapeutics, a plain and easily understood method of healing the sick. It may or may not be explained. Put it to a practical test; that is all we ask." This is pure empiricism, and not science. It only depends upon some facts deduced from practice, and is content with some rules for guidance. It is in no way different from the old practice, which likewise is deduced from certain facts and proceeds according to some rules for guidance derived from them. All empirical knowledge depends upon facts which have been correctly observed. These are collected to a whole of experience, and originate some laws which then are used in practice. This is Dr. Wilson's idea of Homœopathy. The facts are that *similia similibus curantur*. In finding the *similia* on one side upon the sick, the totality of symptoms gives the fact of the pathogenesis, and in finding the *similia* upon the healthy, the totality of the symptoms produced in the healthy gives the fact of the pathopoesis. Both neutralize each other when the remedy is given in the proper dose, and that is the fact of the cure. From these facts the rule *similia similibus curantur* is deduced. And that is all there is of Dr. Wilson's empiricism. It is only a step to science; only the material furnished to lead by induction to the science of healing. Now, Dr. Wilson is so down upon theory, and yet even his empirical view is nothing but a theory. What is a theory? An explanation of facts according to general laws, and it is not such a contemptible thing as many make it out to be. When Dr. Wilson, from the facts of experience, experiment, and observation, concludes that *similia similibus curantur*, he has found the general law governing the cure; this conclusion is the theory of his empirical experience, and so far his proceeding is perfectly justified.

But it is not enough to have reached a sound theory by deduction from the facts, and a rational cognition of the same; it must, if true, depend upon the double proposition :

First, That the empirical matter of fact and the general laws are thoroughly understood, and

Second, That the realm of the rational knowledge from which a series of experiences are explained is determined correctly.

It would lead too far to show how short Homœopathy comes in understanding the empirical data and the general laws thoroughly when Dr. Wilson himself rejects the vital force of the organism.

*As scientists, we take matter and force as our ultimates, etc.*

The pretension that chemistry and physics are the only objects of science, must be rejected. They are sciences, and so there are other sciences, and the science of healing is one of them. But of course, from the limited empirical standpoint, Dr. Wilson cannot yet discern any science of Homœopathics,* though it has all the attributes of a science. It has a multitude of well-established facts connected by a general principle which is in accordance with the laws of motion, because they themselves are one application of it.

If the scientists of the physico-chemical school are satisfied with their prime principle of matter and force which is sufficient for them to explain chemical and physical facts, it does not follow that matter and force are ultimates of other scientists of other schools—for instance, of the biological school. The hope held out, that the principle of the physico-chemical school will as it progresses be able to explain even what biologists call the vital force, is one not to be entertained at the present time, and dies out when we see how insufficient are the results of the investigations in this direction. And it is an impossible undertaking, since life does not originate from matter, but matter is animated by life.

But Dr. Wilson gives no proof for his assertion that "Homœopathy stands upon chemistry and physics with no fear of falling." According to Hahnemann, it stands upon quite a different foundation—the laws of life. The flimsy deduction from the conservation of forces and indestructibility of matter cannot account for the phenomena of life, may the advocates of the agnostic philosophy say ever so much. If force and matter are related to each other as cause and effect, it does not give any information about the origin of force. If force is matter in motion, and matter in motion is the cause of force, and matter in motion is the effect of force, this is a circle of reasoning out of which no life can be constructed. It only is a *testimonium*

*Dunham's Science of Therapeutics.

*paupertatis* which leads to agnostics, *i. e.*, the science of the unknown—a negative science, which can only have a negative effect in denying what is as open as day to every thinking mind, if not biased by chemistry and physics.

*We reject the assumption of a life principle as an unscientific theory.*

This is of no value if Dr. Wilson takes chemistry and physics to be the only science in existence and its votaries the only scientists in the world. The vital force is a fact which every birth and every death and daily life can testify to. But it is useless to argue with people who do not want to acknowledge themselves beaten. How can Dr. Wilson say that it is not necessary to account for the phenomena of health and disease? How can he, if he takes the totality of symptoms to be the whole disease that is for us possible to know, and these symptoms mostly subjective, and of the greatest value in the healing art? How will chemistry and physics come to our aid if a person is insane and there is no chemical and physical data there to go upon? And will chemistry and physics explain the action of Hahnemannian high potencies in which chemical and physical tests of the greatest delicacy cannot discern the least thing? Nay, the overbearing of these late daughters of philosophy is intolerable. They fail to show the vital phenomena to be the consequences of matter and force, and fall into the apotheosis of ignorance, giving it the name of Agnostics, and the host of those who are innocent of proficiency in these otherwise so useful branches of science accept their false philosophy, and pride themselves upon their knowledge gained by others.

*And more especially we object to making it the foundation of the homœopathic healing art.*

Dr. Wilson comes too late. Hahnemann has done so and with perfect right. If Dr. Wilson deviates from him in this vital point, and falls back upon a mere empirical standpoint, he only shows the retrogression which the modern exact sciences have inflicted upon the homœopathic profession. That they are the great majority, does not in the least affect the truth of the Hahnemannian principle, because every cure with a high potency proves him to be correct. Why the truth of a vital force should be shadowy and untenable is easier said than proved, when all the phenomena concerning health and disease testify to it. If the ingrown toe-nail is cured by the thousandth centesimal potency of Graphites again and again, what explanation can chemistry and physics offer for it? It is accidental, they say.

But if the proving of Graphites proves the cure to be homœpathically correct, where is, then, Dr. Wilson's agnostic philosophy?

Dr. Wells is right. Without vital force as a chief factor, Homœopathy has no philosophy, as Dr. Wilson himself proves by assuming the empirical standpoint. But Dr. Wilson does not want a rational philosophy, such a one as founded upon the reason of man. He wants one made to order of the scientists of to-day, and says we have such a one. We do not know of it; Dr. Wilson will have to produce it in order to make good his claim.

To contemptuously put the vital force among the superstitions of old is not the scientific expression of a philosopher but rather a *ruse* of a beaten antagonist. If chemistry and physics have not arrived yet at the truth of a vital force, it is not to be wondered at, for who can expect from these merely physical sciences the solution of the greatest problem that ever was presented to man, the action of immaterial life upon the forces of matter?

*Ceterum censeo, macrodosiam esse delendam.*

